# Supplementary material for: Adherence to and experiences of K–12 students in modified and standard home quarantine during the SARS-CoV-2 pandemic in Missouri
Source: PLoS One. 2023 Jan 17;18(1):e0275404. doi: 10.1371/journal.pone.0275404 (PMC9844916; doi:10.1371/journal.pone.0275404)
Supplement: S1 Appendix — (DOCX) [file pone.0275404.s002.docx]

Appendix: Coding structure for qualitative thematic analysis of parent responses to the open-ended question

| **Please share any details about how you and your family were most affected by quarantine.** | | |
| --- | --- | --- |
| **Code** | **Definition** | **Resulting Theme** |
| Student - Isolation/Anxiety | This includes the anxiety and isolation of quarantine on the student, focusing on isolation from peers and activities | Negative mental health impacts |
| Student - Frustration/Anger | This includes the frustration or aggression experienced by the student resulting from quarantine. |  |
| Student - Depression | This is greater than simply isolation/anxiety, but represents a larger emotional struggle or reported depression |  |
| Student - Extracurricular | Students missing extracurricular or stress surrounding extracurricular involvement during quarantine; note any themes (sports, etc) | Impact on child's activities |
| Student - Work | Students who are working and could not go to their job during this time |  |
| Student - Virtual School | Students having to participate in virtual school (stress, coordination, experiences surrounding schooling) | Educational impacts |
| Student - Missed School | Parents and students expressed concern around missing school days, being out of school |  |
| Student - Screen time | Concerns by parents or students about an increase in screen time/online time; note screen time due to school vs. tv/video games/etc not related to schooling |  |
| Student - No Impact | This describes the lack of impact quarantine had on the student or the student's family. | Limited or positive impacts of quarantine (student) |
| Student - Positives of Quarantine | This captures the good things about quarantine (i.e. getting to sleep in, time together, etc) |  |
| Parent - Work | Parents talk about balance of paid time off, having to miss work, re-arranging work during their student's quarantine | Effects on parent's work |
| Parent - Decision Making | Different decisions that parents had to make and anything they had to take into consideration, this might also include the emotions that parents describe around having to make some of these choices | Decision making around quarantine |
| Parent - Positives of Quarantine | This is for positives for parents - things such as having less to do, more time together, etc | Positives of Quarantine (parent) |
| Parent - Separation in the home | Parents described the stress of having to separate in the household, the stress of having to divide family and make choices about how to remain safe | Negative impact on parent/family's mental health |
| Parent - Frustration/Anger | Parents described anger or frustration around their child being in quarantine or the effects of quarantine |  |
| Family - Isolation | Characterized as separation from family members, such as grandparents |  |
| Family - Fear of illness | This is about fear/anxiety of getting COVID-19 or spreading COVID-19 |  |
| Family - Testing | Availability of tests and having testing available to ease people's concern | Concerns around testing |
